# Continuous determination of blood glucose in children admitted with malaria in a rural hospital in Mozambique

**DOI:** 10.1186/s12936-017-1840-x

**Published:** 2017-05-02

**Authors:** Lola Madrid, Antonio Sitoe, Rosauro Varo, Tacilta Nhampossa, Miguel Lanaspa, Abel Nhama, Sozinho Acácio, Isolina Riaño, Aina Casellas, Quique Bassat

**Affiliations:** 1 Centro de Investigação em Saúde de Manhiça (CISM), Maputo, Mozambique; 2 ISGlobal, Barcelona Ctr. Int. Health Res. (CRESIB), Hospital Clínic-Universitat de Barcelona, Rosselló 132, 5-2ª, 08036 Barcelona, Spain; 3 AGC Pediatria Hospital Universitario Central de Asturias, Oviedo, Spain; 4 Ciber de Epidemiología y Salud Pública (CIBERESP), Madrid, Spain; 5 ICREA, Pg. Lluís Companys 23, 08010 Barcelona, Spain

**Keywords:** Hypoglycaemia, Blood glucose, Hyperglycaemia, Continuous glucose monitor, Malaria

## Abstract

**Background:**

Hypoglycaemia is a frequent complication among admitted children, particularly in malaria-endemic areas. This study aimed to estimate the occurrence of hypoglycaemia not only upon admission but throughout the first 72 h of hospitalization in children admitted with malaria.

**Methods:**

A simple pilot study to continuously monitor glycaemia in children aged 0–10 years, admitted with malaria in a rural hospital was conducted in Southern Mozambique by inserting continuous glucose monitors (CGMs) in subcutaneous tissue of the abdominal area, producing glycaemia readings every 5 min.

**Results:**

Glucose was continuously monitored during a mean of 48 h, in 74 children. Continuous measurements of blood glucose were available for 72/74 children (97.3%). Sixty-five of them were admitted with density-specific malaria diagnosis criteria (17 severe, 48 uncomplicated). Five children (7.7%) had hypoglycaemia (<54 mg/dL) on admission as detected by routine capillary determination. Analysing the data collected by the CGMs, hypoglycaemia episodes (<54 mg/dL) were detected in 10/65 (15.4%) of the children, of which 7 (10.8%) could be classified as severe (≤45 mg/dL). No risk factors were independently associated with the presence of at least one episode of hypoglycaemia (<54 mg/dL) during hospitalization. Only one death occurred among a normoglycaemic child. All episodes of hypoglycaemia detected by CGMs were subclinical episodes or not perceived by caregivers or clinical staff.

**Conclusions:**

Hypoglycaemia beyond admission in children with malaria appears to be much more frequent than what had been previously described. The clinical relevance of these episodes of hypoglycaemia in the medium or long term remains to be determined.

## Background

Hypoglycaemia is a common problem in paediatric emergency admissions in Africa, and is associated with a wide variety of disorders and diseases. In descriptive studies conducted in different countries of Africa, the prevalence of hypoglycaemia on admission ranged between 3.2 and 7.3% of the paediatric patients [1–6]. The plasmatic glycaemia threshold defining hypoglycaemia has been established by the World Health Organization (WHO) as <2.5 mmol/L (45 mg/dL) in an adequately-nourished child and <3 mmol/L (54 mg/dL) in a severely malnourished child [7], however, an increased risk of death has been associated to intermediate levels of low glycaemia [8] and the WHO recommends correction when blood glucose <3.0 mmol/L is detected. Most literature on childhood hypoglycaemia links the incidence of hypoglycaemia to specific conditions such as malaria, diarrhoea or malnutrition, and other life-threatening diseases including meningitis and sepsis as causes that contribute to its occurrence and outcome [1–4, 9, 10]. Hypoglycaemia can complicate many childhood diseases being a frequent albeit-treatable cause of death. It is also a factor of poor prognosis in admitted children [11].

In patients with malaria, decreased levels of glycaemia are common and secondary to the consumption of glucose by the *Plasmodium* parasite, hyperinsulinism caused by quinine (whenever used), and lack of adequate supplementation/oral intake in cases of severe malaria, especially in cerebral malaria [9, 10, 12, 13]. Its impact can be easily understood when assessing the mortality risk in patients with malaria. Indeed, mortality in severe malaria may increase from 8 to 13.4% in patients with normal blood glucose levels to 24–61.5% in the hypoglycaemic patients [8, 14, 15].

The studies conducted to date in children admitted with malaria performed their occasional glycaemia assessments using glucometers that need a finger prick each time the glucose level must be assessed, or relied on the analysis of venous blood samples. These methods only allow limited number of glycaemia assessments during the day. However the evolution of blood glucose is dynamic and hypoglycaemia may occur and be undetected in severely ill patients in the time lapse between two assessments, even after presenting normal blood glucose levels on admission.

To improve monitoring of blood glucose, continuous glucose monitors (CGMs), initially developed for a more timely control of adult and paediatric patients suffering from type 1 diabetes, [16, 17], have been recently introduced in paediatric intensive care, where they are showing a high degree of agreement with standard glucometers [18–20].

Using the CGMs during the initial and most critical days of admission, could allow a better detection of hypoglycaemia episodes, and a comprehensive description of the dynamics and evolution of blood glucose. This would help establishing some preliminary risk factors for hypoglycaemia during the first days of illness, and help to detect those patients who could most benefit from additional supplementation and a more thorough control to improve prognosis during hospitalization.

A pilot study was conducted using CGMs to continuously assess blood glycaemia and aimed to determine the occurrence of hypoglycaemia episodes detected by these devices in children admitted with malaria, trying to identify risk factors associated with the development of intra-admission hypoglycaemic episodes.

## Methods

### Study site and population

This study was conducted in Manhiça, southern Mozambique. Manhiça is a semi-rural, savannah setting with a predominantly young population (19.1% are <5 years of age) [21]. Seasonality is marked, including a hot and rainy season (November–April), which coincides with the peak transmission season for malaria, and a dry and cooler season during the rest of the year.

The Manhiça Health Research Center (CISM) is a leading research centre in Africa with a well-developed clinical laboratory supporting the Manhiça District Hospital (MDH). The CISM runs a demographic surveillance system linked to a morbidity surveillance platform established at the Manhiça district Hospital and five additional primary health centres. A detailed description of CISM and the study area can be found elsewhere [21].

### Study design

This prospective pilot study, conducted at the Manhiça District Hospital during a period of 9 months (1st September 2013 to 31st May 2014), recruited paediatric patients diagnosed with malaria in order to estimate the real occurrence of hypoglycaemia not only upon admission but also throughout the first 72 h of hospitalization. Children aged ≤10 years and fulfilling criteria for admission because of malaria diagnosis and living in the study area, were eligible to be included in the study if guardians agreed.

### Hospital morbidity surveillance system

Hospital surveillance data are routinely collected for all children less than 15 years old visiting the outpatient clinics in the study area and those admitted to the MDH. Clinical data, including medical history, physical examination, routine laboratory basic investigations, ICD-10 based diagnosis, outcome and medication prescribed are collected on questionnaires on a round the clock basis and reviewed daily by senior medical staff before being entered in specific databases. On admission, a finger-prick blood sample is collected to determine PCV and blood glucose concentration. In order to quantify *Plasmodium falciparum* parasitaemia, thick and thin blood films are prepared, processed and double read in the CISM laboratory according to standard procedures [22]. Slides are first read using the ‘Crosses system’ [23] to guide case management. Blood cultures are systematically performed for all children under 2 years of age and, in older children, in the presence of severe symptoms or according to the admitting clinician’s call.

HIV status information is not routinely collected. An HIV rapid diagnostic test or other molecular diagnostic methods for those <18 months of age is performed to those children with suspected HIV infection.

### Definitions

Malaria is defined at MDH as a positive slide regardless of parasitaemia. In order to increase sensitivity and specificity of malaria case definition, we used an age cut-off for the level of parasitaemia based on previous malariometric indicators published for the Manhiça District [24, 25]. Thus, for children aged 12 months or more, a malaria case was defined as a positive blood slide with at least 2500 parasites/µL together with the documented presence of fever (≥37.5 °C, axillary) or a history of fever in the preceding 24 h. For infants (<12 months), no minimum parasitaemia was required. Severe malaria was defined following the WHO severe malaria latest guidelines [26]. Different thresholds were used to define blood glucose levels based on local and WHO guidelines [7] and the increased risk of mortality associated to intermediate levels of low glycaemia [4, 8, 15]: (1) severe hypoglycaemia was defined as glycaemia <45 mg/dL (2.5 mmol/L) in a well-nourished child or <54 mg/dL (3.0 mmol/L) in a malnourished child, detected through the use of the continuous glucose monitoring device and/or a conventional portable bedside glucometers [7]. (2) Any hypoglycaemia implied glycaemia <54 mg/dL (3.0 mmol/L) in well-nourished children [7]. (3) Normal-low range glycaemia, defined as glycaemia >54 and <90 mg/dL (3–5 mmol/L). (4) Subclinical hypoglycaemia was defined as any hypoglycaemia episode (as defined above) not accompanied with apparent clinical symptomatology, as confirmed by the patient or described by the observing clinician. Children were not continuously under medical observation and some episodes categorized as subclinical could be “non-witnessed” by clinical staff. (5) Hyperglycaemia was defined as a glucose value ≥198 mg/dL (11.0 mmol/L) [7]. Fasting was defined according to American Diabetes Association (ADA) criteria [27]. Weight-for-height Z-scores (WHZ) were calculated for each child <5 years old and body mass index for age for children aged over 5 years, using the WHO growth chart [28]. The types of acute malnutrition were defined as follows: global acute malnutrition (<−2 z-score and/or oedema), severe acute malnutrition (<−3 z-scores and/or oedema) or non-existent (Z-score >−1). Dehydration was defined according to WHO guidelines [23].

### Study procedures

Recruitment occurred during the working hours (07.00 am–07.00 pm) in the “Short stay admission” ward (*Internamento de curta duração*, ICD), where trained people were working. Recruitment was based on the first reading of the blood slide and all children with presence of parasites, fulfilling inclusion criteria when the devices were available were asked to participate in the study. A standardized study specific questionnaire was completed at the moment of recruitment. Demographic, nutritional and clinical data such as vital signs and anthropometry were also recorded. Medication, food and liquids other than water intakes, and events such as vomiting and diarrhoea were daily recorded during the time the CGM was inserted.

### Laboratory procedures

An initial bedside capillary determination of glycaemia was performed on admission as routine management of admitted patients at MDH. Blood cultures were performed as part of the routine microbiological surveillance of admitted paediatric patients ongoing in MDH. A slide for malaria diagnosis was performed. No other samples were routinely obtained. HIV determination was offered to any study patient suspected of being immunocompromized.

### Specific procedures for the continuous measurement of blood glucose

We aimed to use the CGMs (iPro2, Medtronic Iberica^®^ SA, Madrid, Spain) for continuous glucose monitoring for 72 h starting upon admission. Children enrolled were connected to the Medtronic^®^ devices after the signature of an informed consent (IC) by the child’s guardian. The CGMS is composed by the ENLITE sensor (glucose sensor) and the Mini Link transmitter (wireless transmitter), which does not offer real-time information.

The study personnel were trained in the use of the devices. Blood glucose levels in real time as well as calibration of CGM data were performed by taking a capillary blood sample (standard capillary assessment) every 6 h (4 times a day). Correction of glucose levels was performed when necessary following WHO guidelines with an intravenous bolus of 5 mL/kg of 10% dextrose which has been the standard of care at MDH(7).

Values recorded through the continuous monitoring software were downloaded via Care Link™ after sensor removal. Data obtained with the CGMs were compared with those obtained by capillary blood glucometer (CBG). We preferred a finger-prick method, as a comparison to CBG, rather than the more accurate laboratory method because the first is the most used in clinical practice at the MDH.

### Statistical analyses

Statistical analyses were performed using StataCorp. 2015. Stata Statistical Software: Release 14. College Station, TX: StataCorp LP. Descriptive statistics were provided for all variables in the dataset, including frequency tabulations for binary/categorical variables and mean and standard deviation for normal distributed continuous variables and median and interquartile range (IQR) for non-normal continuous variables.

Firth logistic regression was used in order to address issues of separability, small sample sizes and bias of the parameter estimates. Only children fulfilling malaria criteria according to our study definition were included in the analysis. A multivariate logistic regression with penalized likelihood analysis was performed among children fulfilling the age and density-specific malaria diagnosis criteria to assess risk factors for having hypoglycaemia during the admission measured by the CGM, including those variables which showed an association with having hypoglycaemia in the univariate analysis, based on their size of effect (OR <0.5 or >1.5). Statistical significance was set at a *p* value of <0.05 and Confidence intervals (CI) at 95% level.

Paired glucose readings from the continuous glucose monitor (when available) and the standard capillary assessment (from calibrations and hypoglycaemia confirmations) were compared using a modified Bland–Altman analysis to measure agreement with repeated measures and a Clarke error grid analysis (CEG). The CEG identified five areas with different error in accuracy combined with the severity of clinical consequences:

Region A: values within 20% of the reference sensor.

Region B: values outside 20% of the reference sensor, but that would not lead to an inappropriate treatment.

Region C: values leading to an unnecessary treatment.

Region D: values indicating a potentially dangerous failure to detect hypo- or hyper-glycaemia.

Region E: values that would confuse treatment of hypoglycaemia for hyperglycaemia, and vice versa.

These graphs were produced using R [29].

## Results

**Fig. 1:**
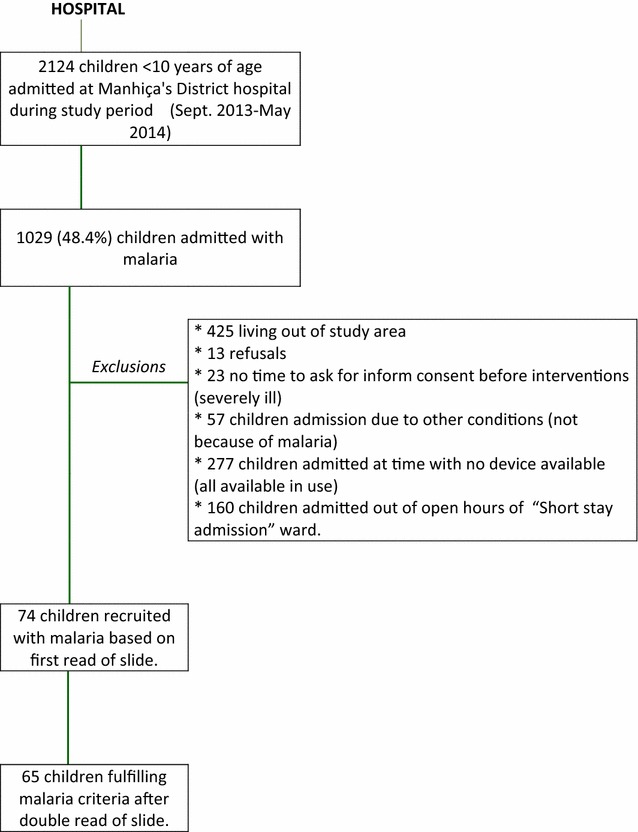
Study profile

A total of 74 children with a positive blood slide in the first reading and admitted because of malaria at MDH were enrolled in the study from September 2013 to May 2014 (study profile in Fig. 1).

Median (interquartile range, IQR) age on admission was 31.5 (5–84) months. Thirty-three children of 74 were male (44.6%) and 22/74 (29.7%) fulfilled the criteria for being considered severe malaria cases. Causes of admission of children recruited in the study are summarized in Table 1. Glycaemia results at admission were available for all children and continuous measurements of blood glucose were available for 72 of them (97.3%) as the CGMs failed to record glycaemia readings in two children. When double reading of parasitaemia was available, seven children were found not to meet density-specific malaria diagnosis criteria. These children were excluded from the analysis, which was therefore restricted to 65 children.

**Table 1:** Cause of admission of children participants of the study

Cause of admission	Uncomplicated malaria N = 52, n (%)	Severe malaria N = 22, n (%)
Prostration	9 (17.3)	NA
Vomiting	7 (13.5)	NA
Difficulty to drink	4 (7.7)	NA
Hyperparasitaemia (first read of blood slide)	32 (61.5)	NA
Cerebral malaria	NA	13
Severe anaemia	NA	8^a^
Hypoglycaemia (<2.5 mmol/L)	NA	3

^a^Two of children admitted with severe anaemia had also cerebral malaria

**Fig. 2:**
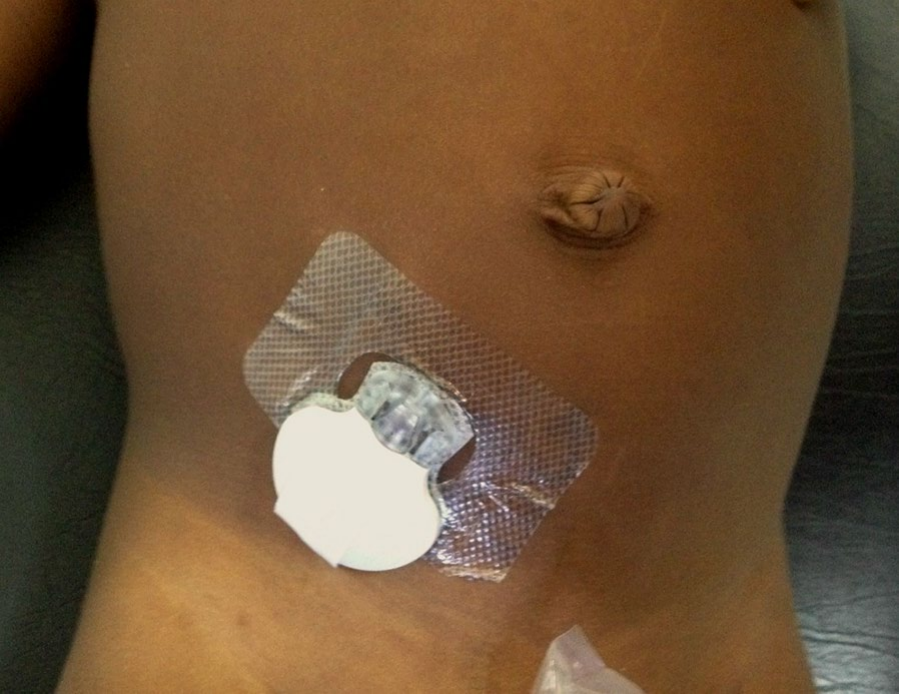
Continuous glucose monitor inserted in a child participating in our study

The devices were well tolerated in all patients and no adverse skin reactions, infections, or bleeding in the insertion site occurred during the study period (Fig. 2). No patient inadvertently removed the device during the study. The average (standard deviation, SD) hospital stay was 2.6 days (0.2). One child with cerebral malaria died.

### Glucose levels at admission measured by capillary assessment

On admission, blood glycaemia was measured by capillary assessment. Five of sixty-five (7.7%) children presented hypoglycaemia <54 mg/dL and three of these episodes (4.6%) were categorized as severe (<45 mg/dL). Hyperglycaemia was detected in 1/65 (1.5%) patients.

### Glucose levels measured by CGMs

Analysing the data collected by the CGM, hypoglycaemia (<54 mg/dL) was detected in 10 of the 65 children (15.4%). Seven of these ten hypoglycaemia episodes (70.0%) were categorized as severe (<45 mg/dL). Six of seventy-two (8.3%) had recurrent (more than one) hypoglycaemia (<54 mg/dL) episodes detected by the CGM. All episodes of hypoglycaemia <54 mg/dL detected by CGMs were subclinical or “non-witnessed” episodes with no development of further complications. Mean (SD) time between CGMs insertion and sensor activation was 82.1 (9.3) minutes. Hypoglycaemic episodes (<54 mg/dL) were nocturne in 5/10 children with hypoglycaemia (50.0%) and the mean (SD) duration of fasting preceding the hypoglycaemic episode was 5.5 h (1.2). Hyperglycaemia was detected in 11/65 (15.9%) of the patients, occurring in 4/10 (36.4%) in the first 2 h after food ingestion.

Table 2 compares some key characteristics upon admission in children with any level of hypoglycaemia with those who remained normoglycaemic throughout their entire hospitalization, as determined by CGMs. Univariate analysis showed median age was not significantly different between the hypoglycaemic (<54 mg/dL) and normoglycaemic groups (39, vs 27 months, respectively, OR 1.01 (0.99–1.03), p = 0.514). None of the risk factors assessed in the univariate analysis appeared to be significantly associated with having more hypoglycaemia (any level) during the hospital admission. The majority of children (32/47) were fasting during the night. One of the ten children (10%) who had a hypoglycaemic episode determined by CGMs during hospitalization also had presented with hypoglycaemia upon admission. Higher axillary temperature was significantly different between those with normal glycaemia and those with at least one hypoglycaemia episode (<54 mg/dL) in the univariate analysis. No difference in duration of hospital stay was found. Only one normoglycaemic child died as compared to none of the hypoglycaemic children.

**Table 2:** Risk factors associated to hypoglycaemia among patients admitted with a malaria diagnosis, according to the univariate analysis

	Normoglycaemia N = 55, n (%)	Hypoglycaemia N = 10, n (%)	OR^e^ and 95% CI	p value^e^
Socio-demographic characteristics				
Age in months (median, IQR)^a^	27 (10–65)	39 (32–65)	1.01 (0.99–1.03)	0.514
Male gender	30 (54.6)	7 (70.0)	1.79 (0.45–7.07)	0.405
Current breastfeeding	21 (38.2)	2 (20.0)	0.47 (010–2.14)	0.330
History of the current disease				
History of fever	55 (100.0)	10 (100.0)	1.00	
History of cough	16^d^ (33.3)	4 (40.0)	1.36 (0.36–5.21)	0.650
History of vomit	7^d^ (14.6)	2 (20.0)	1.63 (0.33–8.14)	0.553
History of diarrhoea	7^d^ (14.6)	1 (10.0)	0.87 (0.13–5.78)	0.889
Difficulties to breastfeed/anorexia	23 (41.8)	3 (30.0)	0.65 (0.16–2.56)	0.533
Difficulties to drink	11 (20.0)	2 (20.0)	1.14 (0.24–5.37)	0.870
Fasting ≥8 h	37 (67.3)	6 (60.0)	0.71 (0.19–2.68)	0.616
History of seizures	11^d^ (22.9)	1 (10.0)	0.52 (0.08–3.26)	0.481
Anthropometrics				
Weight in kg^b^	27.1 (2.2)	22.6 (4.8)	1.04 (0.92–1.18)	0.497
Global acute malnutrition^c^	3 (5.5)	1 (10.0)	1.93 (0.18–21.13)	0.585
Severe acute malnutrition^c^	1 (1.8)	1 (10.0)	6.00 (0.32–111.87)	0.172
Symptoms and signs on admission				
Axillary temp. (°C)^b^	38.7^d^ (0.2)	37.7 (0.5)	0.59 (0.35–0.99)	*0.046*
Respiratory rate^b^	36.0^d^ (1.2)	30.9 (1.9)	0.92 (0.82–1.02)	0.111
BCS at admission^b^	4.3 (0.2)	3.9 (0.6)	0.83 (0.56–1.23)	0.358
Respiratory distress	5^d^ (10.4)	0 (0.0)	0.38 (0.02–7.36)	0.520
Dehydration	3^d^ (6.3)	1 (10.0)	2.05 (0.26–15.73)	0.489
Pallor	13^d^ (27.1)	5 (50.0)	2.63 (0.69–10.02)	0.157
Jaundice	1^d^ (2.1)	1 (10.0)	5.00 (0.47–53.38)	0.183
Oedema	0^d^ (0.0)	1 (10.0)	15.32 (0.58–405.11)	0.102
Prostration	11 (20.0)	3 (30.0)	1.81 (0.42–7.51)	0.416
Unconscious (BCS <5)	11 (20.0)	3 (30.0)	1.81 (0.42–7.51)	0.416
Deep coma (BCS <2)	10 (18.2)	3 (30.0)	2.02 (0.48–8.50)	0.336
Investigation				
Malaria diagnosis	55 (100.0)	10 (100.0)	1.00	
HIV infection	2 (3.8)	1 (10.0)	3.25 (0.38–27.63)	0.280
Severe anaemia	5^d^ (10.4)	0 (0.0)	0.44 (0.02–8.56)	0.584
Positive blood culture	1 (1.8)	1 (10.0)	5.74 (0.54–61.11)	0.148
Hypoglycaemia at admission (<3.0 mmol/L)	4 (7.3)	1 (10.0)	1.81 (0.25–13.00)	0.557
Hypoglycaemia at admission (<2.5 mmol/L)	2 (3.6)	1 (10.0)	3.38 (0.40–28.68)	0.264
Normal-low range glycaemia (3–5 mmol/L)	10 (18.2)	1 (10.0)	0.50 (0.06–4.51)	0.529
Hyperglycaemia at admission (≥11.0 mmol/L)	1 (1.8)	0 (0.0)	1.73 (0.07–45.43)	0.742
Glycaemia at admission (mmol/L)^b^	6.3 (0.3)	5.8 (0.6)	0.91 (0.66–1.24)	0.542
Time up to sensor activation (minutes)^b^	82.6 (20.3)	79.3 (32.9)	1.00 (0.99–1.01)	0.857
Hyperparasitaemia (>100,000 parasites/µL)	27 (49.1)	2 (20.0)	0.30 (0.07–1.370)	0.122
Treatment				
Intravenous quinine	1 (1.8)	0 (0.0)	1.73 (0.07–45.43)	0.742
Intravenous artesunate	51 (92.7)	10 (100.0)	1.84 (0.09–36.71)	0.691
Blood transfusion	3^d^ (6.3)	1 (10.0)	0.72 (−1.32–2.76)	0.489
Outcome				
Length of admission in days^b^	2.5 (0.2)	3.0 (0.6)	1.27 (0.84–1.94)	0.259
Severe malaria	14 (25.5)	3 (30.0)	1.33 (0.33–5.43)	0.686
Died	1 (1.8)	0 (0.0)	1.73 (0.07–45.43)	0.742

Value in italic is statistically significant

*BCS* Blantyre coma score

^a^Age is presented as median (IQR)

^b^Variables presented as mean (standard deviation)

^c^Acute malnutrition calculated using weight-for-Height Z score (up to 5 years) or body mass index (>5 years)

^d^These variables contain missing data for 7 patients

^e^OR and p value derived from Firth logistic regression

The multivariate analysis showed no risk factors independently associated with the presence of at least one episode of any level of hypoglycaemia during the admission (Table 3). Four risk factors showed great effect (OR >2 or <0.5), but none were significantly associated with the development of hypoglycaemia during hospitalization.

**Table 3:** Multivariable analysis of risk factors associated with having at least one episode of hypoglycaemia during the admission

Risk factors	Hypoglycaemia N = 10, n (%)	Adjusted OR	95% CI		p value^b^
Oedema^a^	1 (10.0)	20.08	0.74	547.08	0.075
Dehydration^a^	1 (10.0)	2.87	0.36	23.02	0.321
Several acute malnutrition (WHZ <−3DS)	1 (10.0)	6.69	0.60	74.41	0.122
Blood culture positivity	1 (10.0)	6.69	0.60	74.41	0.123

*WHZ* weight-for-height Z score

^a^These variables contain missing data for 7 patients

^b^OR and p value derived from Firth logistic regression

### Agreement between continuous measurements of blood glucose using continuous glucose monitor and blood glucose levels using capillary blood glucometer

**Fig. 3:**
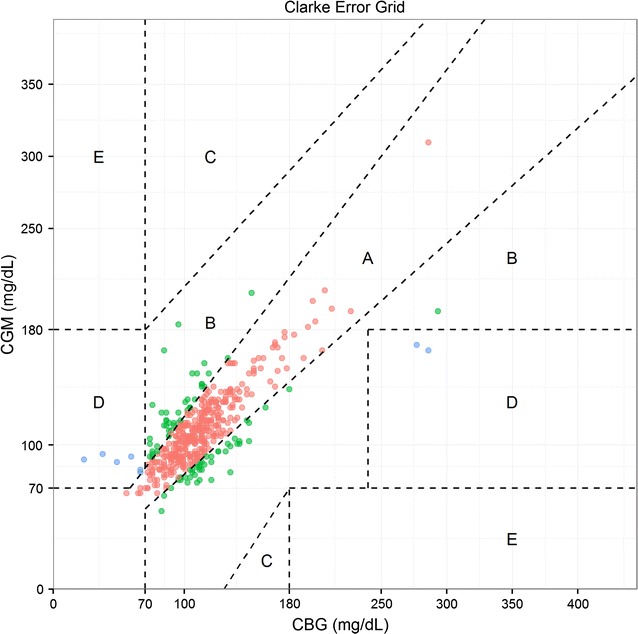
Clarke error grid analysis comparing measures of continuous glucose monitors vs capillary blood glucometer measures. The *figure* is divided in the following 5 regions: *Region A* values within 20% of the reference sensor; *Region B* values outside 20% of the reference sensor, but that would not lead to an inappropriate treatment, *Region C* values leading to an unnecessary treatment; *Region D* values indicating a potentially dangerous failure to detect hypo- or hyper-glycaemia; *Region E* values that would confuse treatment of hypoglycaemia for hyperglycaemia, and vice versa. *CGM* glucose levels measured by continuous glucose monitors, *CBG* capillary glucose levels measured by a glucometer

We used data from 72 patients where CGM measurements were available to assess agreement between both methods of measuring blood glucose. It was possible to obtain 474 paired CGM-CBG measurements. Figure 3 shows the Clarke error grid analysis, where 79.0% of measurements fell in region A, 19.0% of measurements fell in region B, and 2.0% of measurements in region D, without any value in region C or E.

**Fig. 4:**
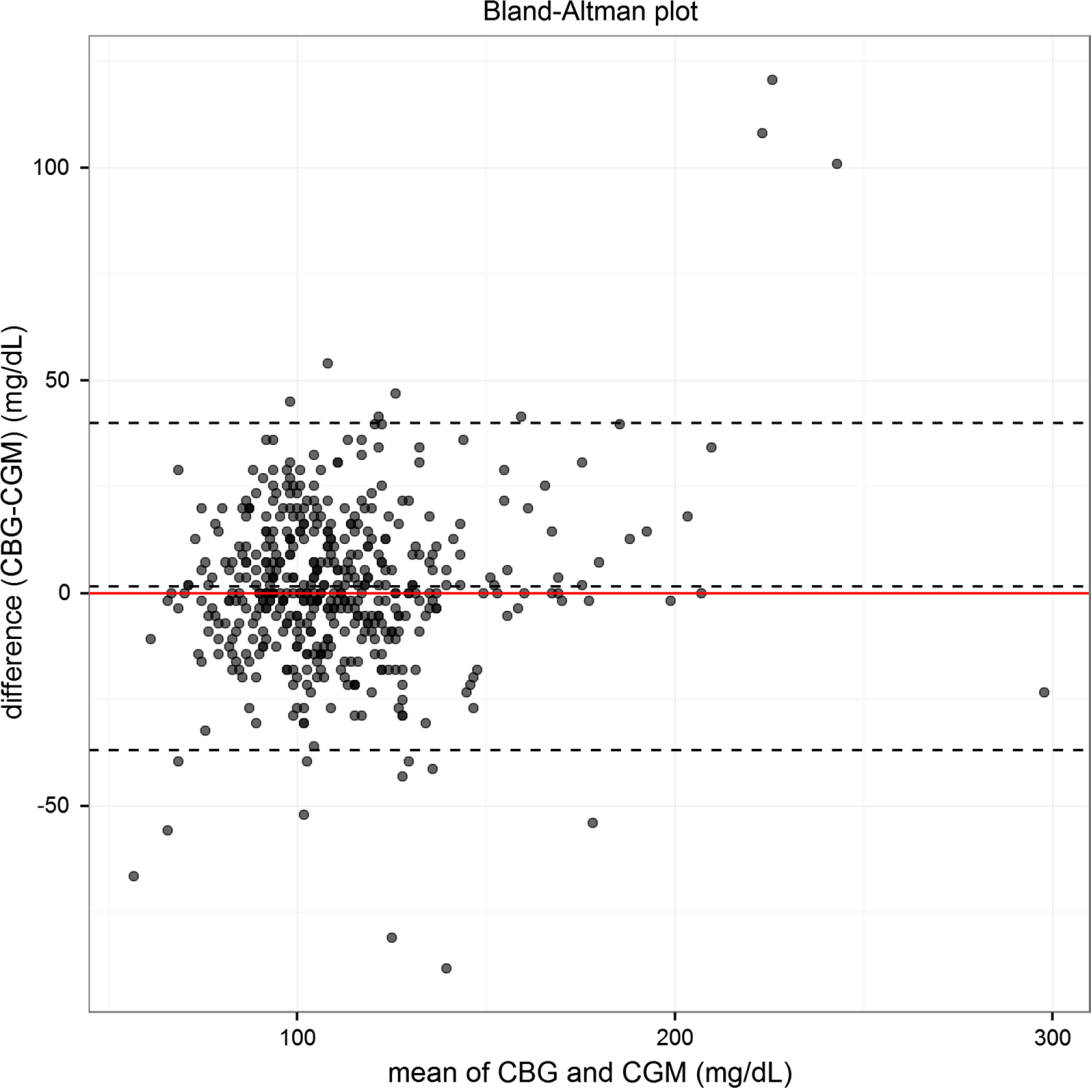
Bland–Altman plot of the difference between glucose levels measured by continuous glucose monitors (CGM) against those measured by capillar determination using a glucometer (CBG). The within-subject variance is estimated by a random effects model. The 95% limits of agreement (−37.0, 40.0 mg/dL) contained 95% of the difference scores

Figure 4 illustrates the Bland–Altman agreement plot. The 95% limits of agreement (−37.0, 40.0 mg/dL) contained 96% (455/474) of the difference scores. The mean difference of the measurements between CGM and CBG methods was 1.5 mg/dL.

## Discussion

This is the first study conducted in a low-income country using CGMs in a rural district hospital. Additionally, it is the first study assessing glucose homeostasis thorough CGMs in children with malaria. Continuous glucose monitoring has been advocated for many years to improve glycaemic management in critical care settings [18, 20], among diabetic patients [30, 31] or among preterm babies [32]. In the present study, the aim was to conduct a descriptive study using these CGMs to continuously assess blood glucose, and eventually detect hypoglycaemic episodes in children with malaria. The prevalence of hypoglycaemia (<54 mg/dL) on admission in this population was 7.7%, more than twofold higher than that found for all-cause admissions in the same setting [4] and similar to that found in other studies in sub-Saharan Africa for malaria inpatients, although hypoglycaemia definition was not uniform across the studies [1–3, 15, 33–36].

A previous study conducted in the same hospital demonstrated that malaria was a strong predictor of hypoglycaemia (OR 1.29, 95% CI 1.09–1.52, p value 0.003) [4]. Since the current study population was restricted to children admitted with malaria, a higher prevalence of hypoglycaemia would have been expected compared to the previous study, which included all-cause admitted children.

Using CGMs, at least one episode of hypoglycaemia (<54 mg/dL) was detected in 10/65 children (15.4%) and in 7/65 (10.8%) using <45 mg/dL threshold, confirming our pre-defined hypothesis that hypoglycaemia in patients with malaria occurs much more frequently than previously described. Only a few studies have explored blood glucose levels beyond admission and throughout hospitalization [15, 36, 37] and using hypoglycaemia thresholds of <54 mg/dL [15] or <45 mg/dL [37], showed a similar prevalence of hypoglycaemia post-admission compared with that found in our study. These studies used standard procedures to measure blood glucose levels such as blood capillary determination [15, 37] and/or venous samples [15, 36, 37]. However, no previous data exist on the occurrence of hypoglycaemia in children with malaria measured with such devices. Only one child with normal-low glycaemia level was detected on admission who did not develop later hypoglycaemia (<54 mg/dL) and survived. This is against to what other authors have reported as an increased risk of later hypoglycaemia and mortality in children with intermediate low glycaemia levels [5, 8, 11]. This difference is likely due to the small sample size and different blood glucose level to define hypoglycaemia.

CGMs were not useful to measure glycaemia at the time of admission, since the sensors started, as expected [38], to detect glucose levels more than 1 h after its insertion, a timing which could make hypoglycaemia consequences become irreversible. Only one child among those with hypoglycaemia on admission presented subsequently hypoglycaemia episodes determined by CGMs. None of the hypoglycaemia episodes were recognised by clinical staff or caregivers, and 50% of these hypoglycaemia episodes were nocturnal. Reasons for this lack of recognition may include, among others, lack of clinical staff at night, poor awareness of symptoms by caregivers or family members, or more simply than those episodes did not associate any clinical symptomatology. Alternatively, it could also be that certain severe conditions, which may last for many hours, including cerebral malaria, may associate clinical but difficult to detect hypoglycaemic episodes in the context of an already altered clinical status. Previous studies using the CGMs have confirmed overnight unrecognized hypoglycaemia is common among children with type 1 diabetes [39] but very little is known on the impact of recognized and unrecognized hypoglycaemia in non-diabetic children. On the other hand, fasting is considered a risk factor because it leads to glycogen depletion, which can result in decreased glucose production and hypoglycaemia [40]. However, using the ADA criteria to measure fasting in our study, no association between lack of caloric intake for ≥8 h and the development of hypoglycaemia could be found. In a study conducted in Suriname, Zijlmans et al. [40] studied glucose kinetics in children with *P. falciparum* malaria during a 16-h period of controlled fasting. During the following 8 h after controlled fasting, plasma glucose concentrations decreased and no episodes of hypoglycaemia were described. In the same study, a linear regression analysis using a mixed linear model was performed and showed children with non-severe malaria would develop hypoglycaemia after 26 h of controlled fasting and the children with severe malaria would develop hypoglycemia after 33 h of controlled fasting (p 0.036) [40]. It is likely that fasting in this study was too short to find any impact on glucose levels in our patients.

Surprisingly, higher axillary temperature was more common among those children whose glycaemia remained normal in the univariate analysis. This finding needs to be interpreted with caution, as no adjustment for multiplicity of testing has been performed. No risk factors independently associated with having at least one episode of any level of hypoglycaemia were found in the multivariate analysis. Caution is however needed when interpreting these results, since sample size was small and likely insufficient to detect significant differences among both glycaemia groups.

Only one child died in this cohort. It has been demonstrated that malaria is a risk factor to develop hypoglycaemia but it is not a predictor of death because of hypoglycaemia [4]. A previous study conducted in the same hospital showed that malaria was not a risk factor associated to mortality in children with hypoglycaemia on admission [4]. However, these findings are restricted to blood glucose levels on admission and there are no data about newly incident or recurrent hypoglycaemia episodes during their continued hospitalization.

The CEG and modified Bland–Altman analyses for all glucose levels demonstrated that the CGM provides similar readings to the CBG. Indeed, 98% of all readings in the CEG were included in the safe A or B regions and there was no evidence of an increase of the measurements into zones (C, D, E), that would lead to inadequate management of hyper- or hypo-glycaemia, as has been previously reported in a study conducted in preterm newborns [32]. The Bland–Altman analysis showed that the 95% limits of agreement contained 96% (455/474) of the difference scores. Previous studies conducted in other populations have shown that CGMs slightly under-reads glucose levels as compared to CBG [32, 41]. This fact may be explained on account of the differences among studied populations (patients with diabetes or newborns *versu*s children with malaria) and/or the use of different glucometers.

The CEG was initially used to test the accuracy of blood glucose measurement in adults or children with diabetes [42]. Two previous studies performed in preterm babies [32, 43] utilized the CEG to compare glucose values obtained from a Medtronic^®^ device with values obtained from different blood glucose tests and found great correlation between the two methods. However, the interpretation of results in a different population and with different interventional glucose levels needs to be cautious [43].

This study has several limitations. First, the study lacked sufficient statistical power to detect significant associations between hypoglycaemia and its determinants, given the small sample size. Second, as the number of devices was limited, recruitment could not be performed continuously, jeopardizing the representativeness of these results. Third, the CGM system has proven adequate and robust for hyperglycaemia determination [44], but its accuracy may be limited for paired glucose recordings during hypoglycaemia [45]. Sensors used in this cohort had a minimum threshold of 2.2 mmol/L and lower glucose levels were detected, albeit with no measurable values. Finally, and as noted above, the sensor may overestimate hypoglycaemia slightly. However, these results confirm a good correlation between CGMs and capillary assessment.

## Conclusions

Although the use of CGM appears insufficiently cost-effective in resource-constrained settings, these data suggest that the CGM is a clinically accurate instrument to evaluate blood glucose levels in children with malaria. Moreover, these data suggest that hypoglycaemic events detected by conventional glucometers may be only the tip of the iceberg of the real burden of hypoglycaemia in children admitted with *P. falciparum* malaria, because many more hypoglycaemia episodes seem to occur, in spite of their poor clinical translation. A higher sample size would be needed to help establishing some preliminary risk factors for hypoglycaemia during the first days of illness, and help detecting those patients who could most benefit from additional supplementation and a more thorough control to improve prognosis during hospitalization.
